# DREAMTools: a Python package for scoring collaborative challenges

**DOI:** 10.12688/f1000research.7118.2

**Published:** 2016-04-08

**Authors:** Thomas Cokelaer, Mukesh Bansal, Christopher Bare, Erhan Bilal, Brian M. Bot, Elias Chaibub Neto, Federica Eduati, Alberto de la Fuente, Mehmet Gönen, Steven M. Hill, Bruce Hoff, Jonathan R. Karr, Robert Küffner, Michael P. Menden, Pablo Meyer, Raquel Norel, Abhishek Pratap, Robert J. Prill, Matthew T. Weirauch, James C. Costello, Gustavo Stolovitzky, Julio Saez-Rodriguez

**Affiliations:** 1 European Molecular Biology Laboratory, European Bioinformatics Institute (EMBL-EBI),Wellcome Trust Genome Campus, Cambridge, UK; 2 Bioinformatics and Biostatistics Hub, C3BI, Institut Pasteur, Paris, France; 3 Department of Systems Biology, Columbia University, New York, USA; 4 Sage Bionetworks, Seattle, WA, USA; 5 IBM, TJ Watson, Computational Biology Center, New York, USA; 6 Leibniz Institute for Farm Animal Biology, Institute of Genetics and Biometry, Dummerstorf, Germany; 7 Oregon Health & Science University, Portland, OR, USA; 8 MRC Biostatistics Unit, Cambridge Institute of Public Health, Cambridge, UK; 9 Department of Genetics & Genomic Sciences, Icahn School of Medicine at Mount Sinai, New York, USA; 10 Institute of Bioinformatics and Systems Biology, German Research Center for Environmental Health, Munich, Germany; 11 IBM Almaden Research Center, San Jose, CA, USA; 12 Center for Autoimmune Genomics and Etiology and Divisions of Biomedical Informatics and Developmental Biology, Cincinnati Children’s Hospital, Cincinnati, OH, USA; 13 Department of Pharmacology, University of Colorado Anschutz Medical Campus, Aurora, CO, USA; 14 RWTH Aachen University Medical Hospital, Joint Research Centre for Computational Biomedicine (JRCCOMBINE), Aachen, Germany

**Keywords:** DREAM, collaborative competition, machine learning, crowdsourcing, systems biology, translational medicine, method evaluation, benchmarking

## Abstract

DREAM challenges are community competitions designed to advance computational methods and address fundamental questions in system biology and translational medicine. Each challenge asks participants to develop and apply computational methods to either predict unobserved outcomes or to identify unknown model parameters given a set of training data. Computational methods are evaluated using an automated scoring metric, scores are posted to a public leaderboard, and methods are published to facilitate community discussions on how to build improved methods. By engaging participants from a wide range of science and engineering backgrounds, DREAM challenges can comparatively evaluate a wide range of statistical, machine learning, and biophysical methods. Here, we describe *DREAMTools*, a Python package for evaluating DREAM challenge scoring metrics. *DREAMTools *provides a command line interface that enables researchers to test new methods on past challenges, as well as a framework for scoring new challenges. As of March 2016, *DREAMTools *includes more than 80% of completed DREAM challenges. *DREAMTools *complements the data, metadata, and software tools available at the DREAM website http://dreamchallenges.org and on the *Synapse* platform at https://www.synapse.org.

**Availability:** *DREAMTools* is a Python package. Releases and documentation are available at http://pypi.python.org/pypi/dreamtools. The source code is available at http://github.com/dreamtools/dreamtools.

## Introduction

Crowd-sourcing has gained considerable attention over the last years as an approach to solve complex problems. A specific variant of crowd-sourcing is based on setting up challenges or collaborative competitions, whereby the scientific community is invited to provide solutions for a given problem. Typically, the challenge organizers withhold a *gold standard* and use it to evaluate the performance of the submissions by comparing the latter to the former. At the end of such an exercise, the organizers perform a meta analysis with the aim of deriving lessons about which type of methods seem to be more suitable, which features seem to be good predictors regardless of the method, etc. Importantly, the challenge’s results remain as a resource for the community representing a snapshot of the state-of-the-art and to aide in further method development and benchmarking.

In the context of computational biology, there have been several of these initiatives, including CASP, CAFA, CAPRI, FlowCAP ^1^, CAGI, and Dialogue for Reverse Engineering Assessment and Methods (DREAM; www.dreamchallenges.org) ^2^. The DREAM challenges started with a focus on the field of biomolecular network inference ^3– 5^ but now cover questions ranging from prediction of transcription factor sequence specificity ^6^, to toxicity of chemical compounds ^7^ and the progression of Amyotropic Lateral Sclerosis (ALS) patients ^8^ or survival of breast cancer patients ^9^. Since 2013, DREAM has partnered with Sage Bionetworks and challenges are hosted on Sage’s Synapse platform. Each challenge has a dedicated project space in Synapse where the description, training data set, gold standard and scoring methodology are provided. The scored predictions are also available on a public leaderboard.

A fundamental step in DREAM challenges, or any other collaborative competition, is to assess how well the different predictions fare against the gold standard. This may seem obvious at first glance; for example, for a question of predicting a set of numbers, one can compute the sum of the squared differences between predicted and observed values, and identify the submission for which this sum is the smallest. However, multiple aspects have to be taken into account such as the fact that often the confidence on the different measured values is not the same, or that the differences between the submissions may or may not be different enough to declare one method superior to the other. Over the years, within the DREAM challenges, these questions have been addressed leading to the generation of multiple scoring methods.

Scoring methods developed by challenge organizers are reported in the publications that describe the challenges, but the corresponding code is typically provided only in pseudo-code or at best as a script in an arbitrary language (R, Python, Perl...) and syntax by different developers leading to a set of heterogeneous code. In addition, templates and gold standards need to be retrieved manually. All of these factors present obstacles to maximize the scientific value of DREAM challenges as a framework for *a posteriori* evaluation of a method’s performance in comparison with those used in the challenges. Similarly, reuse of scoring code for future challenges becomes complicated when at all possible.

To facilitate the *a posteriori* use of the challenges resources by the scientific community, we have gathered DREAM scoring functions within a single software called *DREAMTools* that provides a single entry point to the DREAM scoring functions. We also provide a standalone executable for end-users and the ability to share and re-use existing code within a common framework to ease the development of new scoring functions for future challenges. *DREAMTools* does not provide code to generate the data or to manage leaderboards (which happens within Synapse), but focuses on the scoring functions. Note that organizers interested in setting up automatic scoring and publishing of leaderboards should instead refer to the section “Create a Scoring application” from the Synapse project 2453886. Currently, *DREAMTools* includes about 80% of the past challenges. For a few challenges where integration in *DREAMTools* was not possible, references to external resources are provided.

**Figure 1.**
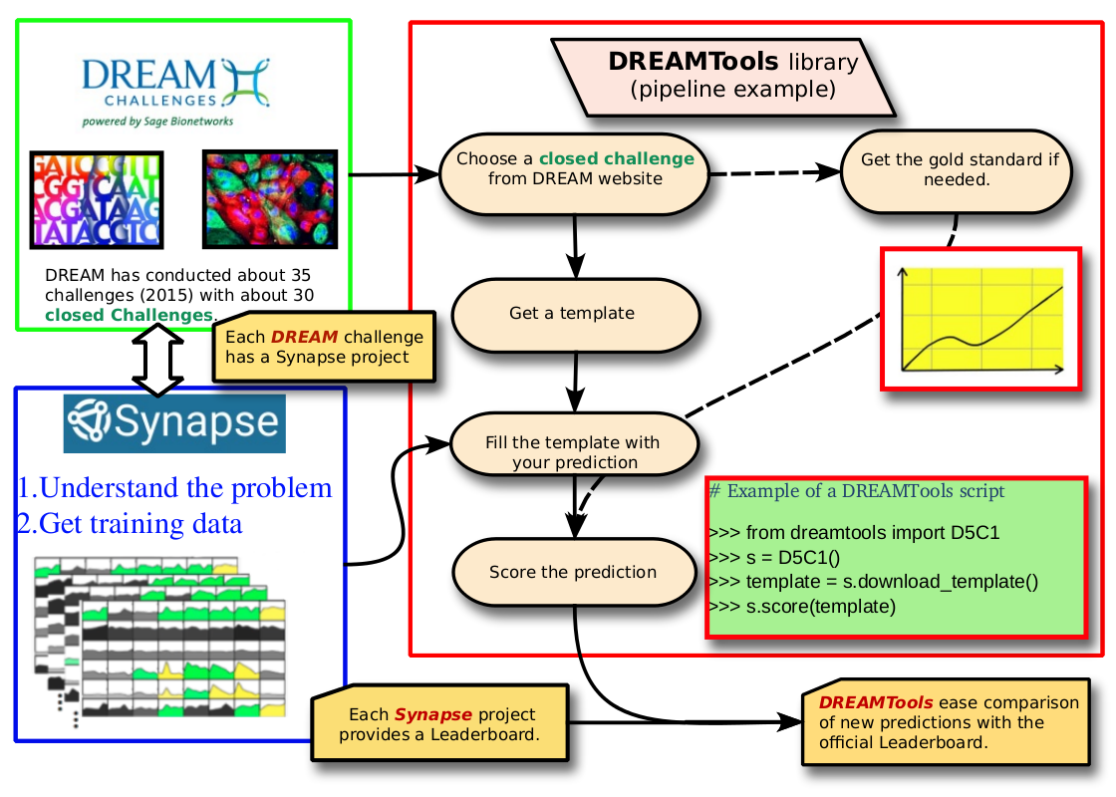
*DREAMTools* library framework. DREAM challenges are described at the DREAM website ( http://dreamchallenges.org) where researchers can get an overview of the past and current challenges. Each challenge has its own project page within the Synapse framework ( http://synapse.org) where details about the challenge are available. The final leaderboard showing benchmarks achieved at the end of the challenge are also shown in the Synapse project. *DREAMTools* provides a Python library that allows researchers to retrieve a template for each closed challenge and to easily score a prediction/template against the gold standard. In a few lines of code, the score of a prediction can then be compared to the official leaderboard, as illustrated in the example in the green box on the right hand side of the figure.

Here, we first describe the framework used in *DREAMTools* software from the point of view of both an organizer/developer and an end-user (see Figure 1). We then review the challenges and the scoring functions that are available until now.

## Methods

The diversity of challenges proposed by DREAM (see Available challenges section) and the plethora of languages that have been used in past challenges has led to a fragmentation of the software designed to score submissions. In order to tackle this problem, we chose Python as a glue language. In addition to a clear syntax and the ability to scale-up software, Python can include compiled codes (e.g., Fortran and C) or call other scripting languages (Perl, R). Besides, languages such as Ruby or MATLAB can also be easily translated to Python, which was an invaluable asset to incorporate many of the earlier challenges that were originally encoded in MATLAB.

*DREAMTools* is an open-source library. Consequently, it can be used directly to evaluate a method against the gold standard of the corresponding challenge, and can also be used as a framework to develop further scoring schemes within the DREAM umbrella or elsewhere.

With about four challenges a year, we use a convention to easily refer to a given DREAM challenge. We decided to closely follow the convention adopted on the DREAM website and use a nickname that takes the form **DXCY**, where *X* is set to the DREAM version and *Y* is set to the challenge number. For example, the HPN-DREAM Breast Cancer challenge ^10^ will be referred to as *D8C1*. If a challenge has sub-challenges, we will also need to provide names to identify them. We do not enforce any convention on sub-challenge names. The nicknames can be found here below in Table 1.

**Table 1.** Availability of the DREAM scoring functions within *DREAMTools*. The first column provides the nickname used in *DREAMTools* to refer to a challenge. The challenge’s title (second column) and its Synapse identifier (fourth column) can be used to retrieve all details about a challenge. The third column gives the challenge status within *DREAMTools*: most of the challenges’ scoring functions are implemented in *DREAMTools* (green boxes); open challenges are not yet available (blue boxes); a couple of challenges did not release the gold standard and may not be implemented (red boxes labelled ’No GS’ for no gold standard); some are to be implemented in future releases (orange boxes labelled ’TBD’ for to be done).

DREAM Nickname	Title	Availability	Synapse ID
D2C1	BCL6 Transcriptional Target Prediction	Implemented	3034857
D2C2	Protein-Protein Interaction Network Inference	Implemented	2825374
D2C3	Synthetic Five-Gene Network Inference	Implemented	3034869
D2C4	*In Silico* Network Inference	Implemented	2825394
D2C5	Genome-Scale Network Inference	Implemented	3034894
D3C1	Signaling Cascade Identification	Implemented	3033068
D3C2	Signaling Response Prediction	Implemented	3825325
D3C3	Gene Expression Prediction	Implemented	3033083
D3C4	*In Silico* Network	Implemented	2853594
D4C1	Peptide Recognition Domain Specificity Prediction	Implemented	2925957
D4C2	*In silico* Network Challenge	Implemented	2925957
D4C3	Predictive Signaling Network Modeling	Implemented	2825304
D5C1	Epitope-Antibody Recognition Specificity Prediction	Implemented	2820433
D5C2	Transcription Factor DNA Motif Recognition	Implemented	2887863
D5C3	Systems Genetics Challenge,B	Implemented	2820440
D5C4	Network Inference Challenge	Implemented	2787209
D6C1	Alternative Splicing	TBD	2817724
D6C2	see D7C1	Implemented	2841366
D6C3	Gene Expression Prediction	Implemented	2820426
D6C4	FlowCAP2 Molecular Classification of Acute Myeloid LeuKaimia	Implemented	2887788
D7C1	Network Topology and Parameter Inference	Implemented	2821735
D7C2	Breast Cancer Prognosis	TBD	2813426 and 1710250
D7C3	The DREAM Phil Bowen ALS Prediction Prize4Life	Implemented	2826267
D7C4	NCI-DREAM Drug Sensitivity	Implemented	2785778
D8C1	HPN-DREAM Breast Cancer Network Inference	Implemented	1720047
D8C2	NIEHS-NCATS-UNC DREAM Toxicogenetics	Implemented	1761567
D8C3	The Whole-Cell Parameter Estimation	TBD	1876068
D8dot5	The Rheumatoid Arthritis Responder	Implemented	1734172
D9C1	The Broad-DREAM Gene Essentiality Prediction	Implemented	2384331
D9C2	Acute Myeloid Leukemia Outcome Prediction	No GS	2455683
D9C3	Alzheimer’s Disease Big Data	No GS	2290704
D9C4	ICGC-TCGA-DREAM Somatic Mutation Calling	TBD	312572
D9dot5C1	Olfactory Challenge	Implemented	2811262
D9dot5C2	Prostate Cancer	TBD	2813558
D10C1	DREAM ALS Stratification Prize4Life	Open Challenge	2873386
D10C2	ICGC-TCGA-DREAM Somatic Mutation Calling Tumor Heterogeneity	Open Challenge	2813581
D10C3	ICGC-TCGA DREAM Somatic Mutation Calling RNA Challenge (SMC-RNA)	Open Challenge	2813589

In this section we provide a brief overview of the scoring functions; for further details we point the reader to the detailed documentation on Read The Docs ( https://dreamtools.readthedocs.org).

### *DREAMTools* for end-users: The dreamtools executable

*DREAMTools* provides a standalone application called **dreamtools**, which is installed with the *DREAMTools* library (see Installation section for details). Note that the application’s name uses lower cases to facilitate the user’s experience. The **dreamtools** application needs a few arguments. The first argument is the challenge name using -- *challenge* followed by the challenge nickname (e.g., D7C2). The second compulsory argument is the filename containing the prediction or submission using the -- *submission* or -- *filename* argument. Some challenges have sub-challenges, in which case an extra argument called -- *sub-challenge* is added. Let us consider the case of the D3C3 challenge (Gene Expression Prediction) ^11^. In order to obtain the score, call **dreamtools** as follows:



                          
  
dreamtools --challenge D3C3 --filename
    template.csv                      
                    


The scoring function of that particular challenge returns a score based on a Spearman rank correlation. Other challenges may return more complex information.

The **dreamtools** standalone application allows one to quickly compute the score of a prediction. However, users or developers may want to script it, which remains concise as shown in the following Python script:



1 # ------ imports the challenge class  
2 from dreamtools import D3C3           
3 # ------ creates an instance          
4 s = D3C3( )                           
5 # ------ retrieves an example         
6 filename = s.download_template()      
7 # ------ scores and prints the results
8 print(s.score(filename))              
                    


**Figure 2.**
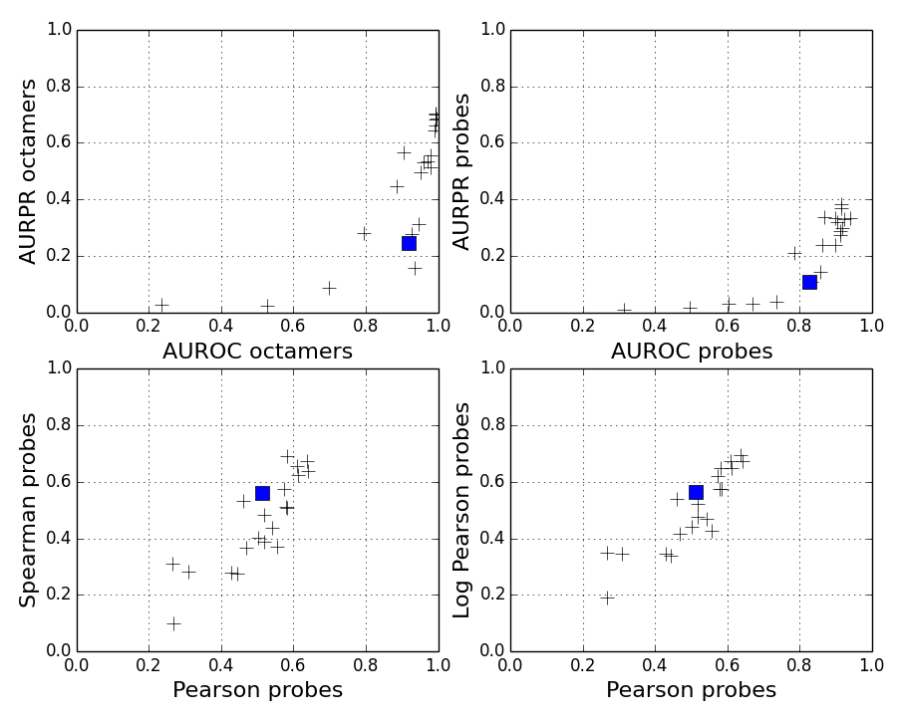
*DREAMTools* provides scoring methods to score or rank new predictions. However, as shown in this figure, other functions may be provided. For instance, the *plot()* method available in the D5C2 challenge shows 4 sub-figures with the score of a submission (blue square) compared to the official participants (black crosses) for 4 metrics (AUROC, AUPR, Spearman versus Pearson correlation). This example is available as an IPython notebook in the *DREAMTools* repository.

Note that all challenges follow the same structure with three main functions: to retrieve a template example, to retrieve a gold standard, and to score a prediction. In addition, **dreamtools** may give access to more functions. For example, the D5C2 challenge ^6^ has a plot method to compare a prediction with the official submissions, that facilitates inspection of the results, as shown in Figure 2. The figure is generated with an IPython notebook ^12^, which is available in the source code repository of *DREAMTools*.

Another useful option from the **dreamtools** executable is the --info option, which provides information such as the title and summary of the challenge but also the list of sub-challenges and the Synapse project page where all details about the challenge can be found:




dreamtools --challenge D8C1 --info
                    


### Templates and gold standards

Most challenges require a gold standard to score a prediction. Small-size gold standards are provided within the library and, to keep *DREAMTools* light-weight, large-size gold standards are stored through Synapse and automatically downloaded when required – using the official Synapse client (see Sec about installation and dependencies). A similar strategy is applied to templates. Users will need to have a login on the Synapse platform to access these files. The downloaded files are stored locally in a standard place (e.g., /home/user/.config/dreamtools directory under Linux systems).

Users can retrieve the location of the templates and gold standards with the **dreamtools** application as follows:



  
dreamtools --challenge D3C1 --download-
    template                           
                    


If sub-challenges are available, a sub-challenge name must be provided. The valid sub-challenge names can be obtained with the --info argument:



  
dreamtools --challenge D8C2 --info
                    


### *DREAMTools* for challenge developers: an easily expandable framework

*DREAMTools* library also provides a framework to ease the addition of other challenges by encouraging the usage of a consistent layout. In order to incorporate a new challenge, a developer can look at previous instances and create manually its own tree structure. However, we provide another standalone application called **dreamtools-layout**. This application requires only one argument: the challenge nickname.



  
dreamtools-layout --challenge D10C10
                    


This command creates a directory named after the challenge nickname. Inside the directory, sub-directories are created to store the templates, gold standards and possibly other data sets. For instance, data to compute p-values may be stored in the *data* directory. Code related to training data generation could be stored in the *generator* directory, and so on.

In addition to the tree directory, some files are created amongst them a *README* file that should be filled with information about the challenge (e.g. Synapse identifier, acronym, summary) and a Python script called **scoring.py**. The basic structure of the scoring script is to provide the same interface for each challenge. In particular, we enforce the implementation of a function to download a template, a function to download a gold standard and a function to score the submission. Here is an example of such a file, which needs to be filled by the developer:


 
1 from   dreamtools import  Challenge       
2 class  D10C10(Challenge):                 
3     def  __init__(self):                  
4         super(D10C10, self).__init__()    
5         self.sub_challenges = [ ]         
6     def score(self, filename):            
7         raise NotImplementedError         
8     def download_template(self):          
9         return path_to_template           
10    def download_goldstandard(self):      
11       return path_to_goldstandard        
                    


Using the code above, the challenge will be automatically available in the standalone application without extra costs to the developer. The *download_template( )* method is not strictly speaking required; it helps a user to create a prediction though and is provided for all challenges. Developers should consider adding tests and documentation in the existing framework. The last release of *DREAMTools* contains a test suite (collection of test cases used to check the software) with a code coverage higher than 80%; it guarantees that the *DREAMTools* functionalities (especially the scoring functions) do work as expected.

### Installation and dependencies

The *DREAMTools* source code is available on GitHub. It can be downloaded and installed as follows:



  
 > git clone git@github.com:dreamtools/  
    dreamtools.git                       
 > cd dreamtools                         
 > python setup.py install               
                    


The source code gives access to the latest version but releases are also provided on the Python repository website ( Pypi) and consequently installation is also possible using the *pip* tool:



  
> pip install dreamtools                
                    


*DREAMTools* relies on established scientific libraries such as Pandas ^13^ for the data mangling, SciKit-learn ^14^ (e.g., ROC curves) and more generally NumPy/SciPy ^15^ for statistical analysis. Those libraries are recognized in the scientific community and there is an ample set of online resources that cover installation procedure.

Yet, the compilation of these libraries may take a while or lead to compilation errors on some machine configuration. Consequently, we also provide a pre-compiled version of *DREAMTools* within the **bio** channel ( http://bioconda.github.io), which is a channel of Anaconda ( https://www.continuum.io/downloads). The latter provides about 400 scientific packages including Numpy and Pandas aforementioned.

Finally, note that in order to keep a light-weight package, we store large data files in Synapse. *DREAMTools* will download files automatically on request. The download is achieved using the Python Synapse client (also available for the R language).

## Available challenges and scoring metrics

*DREAMTools* covers about 80% of the past DREAM challenges, as shown in Table 1. Although there is a wide range of biological problems addressed in the DREAM challenges, most of the scoring functions revolve around a set of established methods. A majority of the challenges are posed as binary classification questions. Here, scoring metrics compare the predictions against a gold standard and derive metrics such as the AUROC (area under the receiver operating characteristic) or AUPR (area under the precision/recall curve). The rest of the challenges are posed as prediction of quantitative values, and use scoring metrics that compare the predicted values and gold standard by computing their correlation, either between the actual values using e.g. Pearson correlation, or between their ranks, using either Spearman’s rank correlation or concordance index (CI).

Some final scores are based on the empirical null distribution of random sets of predictions so that the final scores are p-values. In addition, while scoring metrics such as Spearman’s rank correlation provide an absolute value that can be compared to the leaderboard, in some cases the rank of the prediction when compared to the other participants is also involved in the scoring. In such cases, even if the scores reported in *DREAMTools* use the same scoring functions as those used while the challenge was open, the score reported by *DREAMTools* may be different from what can be found in the published leaderboard.

In this section we provide a short description of each challenge and the scoring metric(s) used. Details about the methods can be found in the Supplementary material (see Section 1). Full details about the data format and scoring metrics for each of those challenges can be found on the dedicated Synapse project, whose identifiers are provided in Table 1. We will use the following conventions whenever possible: the final score (if unique) is denoted *S*. A rank is denoted *R*. A p-value is denoted *p* with a label (e.g., p-value of the AUROC metric is denoted *p*_AUROC_). The gold standard data set is denoted *X* and a prediction from a participant is denoted X^.

### DREAM2

DREAM2 conducted 5 challenges ^16^. The scoring functions are all based on the AUROC and AUPR metrics (see Supplementary material for details).

### D2C1: *BCL6* Transcriptional Target Prediction

**Description:** *BCL6* is a transcription factor that plays a key role in both normal and pathological B cell physiology. The intersection of two independent data sets of transcriptional targets of *BCL6* (based on (i) ChIP-on-ChIP data and (ii) molecular perturbations) provided 53 functional *BCL6* gene targets. In this challenge a set of 147 decoy genes were randomly selected (with no evidence of being *BCL6* targets) and combined with the 53 functional *BCL6* genes to a list of 200 genes in total. The challenge consisted of identifying which genes are the true targets (and the decoys); to do so, participants were given an independent panel of gene expression data ^17^.
**Scoring metric:** Using a binary classifier, the AUPR and AUROC metrics are computed.

### D2C2: Protein-Protein Interaction Network Inference

**Description:** The challenge consisted of determining the set of true positive and true negative protein-protein interactions among all the pairwise interactions possible within a network of 47 proteins (yeast) ^16^.
**Scoring metric:** The list of gene pairs are ordered according to the confidence. Using a binary classifier and a gold standard of gene pairs, the AUPR and AUROC metrics are computed.

### D2C3: Synthetic Five-Gene Network Inference

**Description:** In this challenge, a 5-gene synthetic-biology network was created and transfected to an *in vivo* model organism. Participants were asked to predict the connectivity of this network using *in vivo* measurements. Two slightly different networks were built using quantitative PCR or Affymetrix chips. Each version had 6 variants depending on the nature of the networks (e.g., signed vs unsigned networks).
**Scoring metric:** Each submitted network is scored independently using the AUPR and AUROC metrics.

### D2C4: *In Silico* Network Inference

**Description:** Three *in silico* networks were created and endowed with deterministic dynamics that simulate biological interactions. The challenge consisted in reverse engineering those networks. The first and second networks had about 50 nodes and 100 directed edges with Erdos-Renyi and scale-free topology, respectively. The third network was a full *in-silico* biochemical network with 23 proteins, 24 metabolites and 20 genes through 146 directed edges ^16^. Each network had 5 variants depending on the nature of the networks (e.g., signed vs unsigned networks).
**Scoring metric:** Same as D2C3.

### D2C5: Genome-Scale Network Inference

**Description:** A panel of normalized *E. coli* Affymetrix microarrays were provided. The challenge consisted of reconstructing a genome-scale transcriptional network of 3456 genes and 320 transcription factors ^18^.
**Scoring metric:** Same as D2C3.

### DREAM3

DREAM3 had 5 challenges fully described in 3.

### D3C1: Signaling Cascade Identification

**Description:** Protein concentrations of four intracellular proteins involved in a signaling cascade were measured in single cells by antibody staining and flow cytometry. The task was to identify each of the measured proteins from among the seven molecular species: complex, kinase, phosphorylated complex, phosphorylated protein, protein, phosphatase, and activated phosphatase) ^3^.
**Scoring metric:** The number of correctly assigned protein identities. Final score is the probability of having *k* or more correct predictions as compared to a random assignment.

### D3C2: Signaling Response Prediction

**Description:** The goal of this challenge was to predict the response to perturbations of a signaling pathway in normal and cancer human hepatocytes. There were 2 sub-challenges: (i) prediction of a subset of *phosphoproteomic* data points measured but removed from normal and cancer hepatocytes data sets (ii) prediction of the concentration of the 20 *cytokines* measured but removed from the training data sets ^3, 4^.
**Scoring metric:** The distance between the prediction and gold standard is computed as the normalized squared error *E*:
  $$E=\sum_{i=1}^{N}\frac{{\left({\hat{X}}_{i}-X_{i}\right)}^{2}}{\sigma_{b}^{2}+\sigma_{s}^{2}{\left[X_{i}\right]}^{2}},\;(1)$$
  with *i* a time index, *σ _b_* = 0.1 represents a baseline, signal independent, measurement noise and *σ _s_* = 0.2 represents a signal dependent measurement noise. Finally, a probability distribution for this metric was estimated by simulation of a null model and a p-value reported as the final score.

### D3C3: Gene Expression Prediction

**Description:** Gene expression time course data were provided for four different strains of yeast ( *S. cerevisiae*): one wild type and three mutants ^11^. Participants were asked to predict the relative expression levels for 50 genes (not part of the training data set) at eight time points in one mutant. For each time point, predictions were submitted as a ranked list (with values from 1 to 50 sorted from most induced to most repressed compared to the wild type expression).
**Scoring metric:** Submissions are scored using Spearman’s rank correlation coefficient between the predicted and measured gene expression at each of the eight time points. The same statistic is also computed with respect to each gene across all time points. Thus, two tests of similarity to the gold standard are computed (time-profiles *T* and gene-profiles *G*). P-values are computed using a test for association between paired samples. The final score is:
  $$S=-\frac{1}{2}\log_{10}\left(p_{T}\times p_{G}\right),\;(2)$$
  where *p _T_* and *p _G_* are the p-value for the time-profiles and gene-profiles, respectively.

### D3C4: *In Silico* Network Challenge

**Description:** The goal of this challenge was to reverse engineer a gene network from time series and steady state data. Participants were asked to predict the directed unsigned network topology from the given *in silico* generated gene expression data sets ^3^. There were 3 sub-challenges with different network sizes (10, 50 and 100) for 5 different data sets.
**Scoring metric:** For a given sub-challenge, predictions are required to be ranked edge-list. The 5 data set predictions are assessed based on the AUPR and AUROC and their respective p-values. Intermediate scores are computed using the log-transformed average of the p-values:
  $$S_{\text{AUROC}}=-\frac{1}{N}\sum_{i=1}^{N=5}\left(\log_{10}\left(p_{\text{AUROC},\text{i}}\right)\right)\;(3)$$
  and
  $$S_{\text{AUPR}}=-\frac{1}{N}\sum_{i=1}^{N=5}\left(\log_{10}\left(p_{\text{AUPR},\text{i}}\right)\right).\;(4)$$
  The final score is the mean of those 2 scores.

### DREAM4

### D4C1: Peptide Recognition Domain (PRD) Specificity Prediction

**Description:** Peptide Recognition Domain (PRD) binds short linear sequence motifs in other proteins. Many protein-protein interactions are mediated by PRD. For example, PDZ domains recognize hydrophobic C-terminal tails, SH3 domains recognize proline-rich motifs, and kinases recognize short sequence regions around a phosphorylatable residue ^19^. This challenge consisted of predicting a position weight matrix (PWM) that describes the specificity profile of each of the domains to their target peptides.
**Scoring metric:** PWM predictions are judged exclusively by similarity to the experimentally mapped PWM using the distance induced by the Frobenius norm, defined as the square root of the sum of the absolute squares of its elements:
  $${\left\|A\right\|}_{F}=\sqrt{\sum_{i=1}^{m}\sum_{j=1}^{n}{\left|a_{ij}\right|}^{2}}.\;(5)$$
  In the kinase case, distances for *N* = 3 PWMs are computed using the Frobenius distance. The p-values of those distances are computed based on random PWMs (a random PWM is formed by entries with values identically and uniformly distributed such that each column normalizes to one). Final score is then the log-transformed average of these p-values:
  $$S_{\text{kinase}}=-\frac{1}{N}\sum_{i=1}^{N=3}\left(\log_{10}\left(p_{\text{pval},\text{i}}\right)\right).\;(6)$$
  Similarly for the PDZ and SH3 sub-challenges with *N* = 4 and *N* = 3, respectively.

### D4C2: DREAM4 *In Silico* Network Challenge

**Description:** Similarly to D3C4, the goal of the challenge was to reverse engineer gene regulation networks from simulated steady-state and time-series data. Participants were asked to infer the network structure from *in silico* gene expression data sets ^20^.
**Scoring metric:** See D3C4 challenge scoring metric.

### D4C3: Predictive Signaling Network Modeling

**Description:** Participants were asked to create a cell-type specific model of signal transduction using the measured activity levels of signaling proteins in HepG2 cell lines ^3^.
**Scoring metric:** The score is the sum of squared errors over all the predictions (see Equation 1) for each protein. Then, p-values are computed and the prediction score is defined as $S_{pred}=-\frac{1}{N}\sum_{i}^{N=7}\log p_{i}$ with *p _i_* the p-value for a given protein. The final score being:
  $$S=S_{pred}-r\times {\text{N}}_{\text{e}}\;(7)$$
  with *r* a weight per edge computed as the minimum over all participants of the prediction score divided by edge count and *N _e_* the number of edge in the network (asked on the prompt). The parameter *r* is used to take into account the parsimony of the submitted network.

### DREAM5

### D5C1: Epitope-Antibody Recognition (EAR) Specificity Prediction

**Description:** Antibody-protein interactions play a critical role in medicinal disciplines (e.g., oncology). Ideally, one specific antibody exclusively binds one specific sequence, however, many antibodies bind to a set of related peptides (or even distinct) and do so with different affinities. A key question is to be able to predict common peptide/epitope sequences that can be recognized by human antibodies. In this challenge, a pool of about 7000 epitope sequences containing peptide sequences reactive with human immunoglobulins was experimentally identified ^21^ to constitute the positive set. Conversely, about 20,000 peptides showed no antibody binding activity and constituted the negative set. Given a training set, the challenge consisted in determining whether each peptide in the test set belongs to the positive or negative set.
**Scoring metric:** The AUROC and AUPR metrics are computed. Their p-values are obtained from null distributions. The overall score is:
  $$S=-\frac{1}{2}\left(\log_{10}\left(p_{AUROC}\right)+\log_{10}\left(p_{AUPR}\right)\right).\;(8)$$

### D5C2: TF-DNA motif Recognition Challenge

**Description:** Transcription factors (TFs) control the expression of genes through sequence-specific interactions with genomic DNA. Modeling the sequence specificities of TFs is a central problem in understanding the function and evolution of the genome. In this challenge, binding preferences of 86 mouse TFs were provided in the form of double-stranded DNA probe intensity signals from protein binding microarrays ^22^. A training data set of 20 TFs was provided and the challenge consisted of predicting the signal intensities for the remaining TFs ^6^. Note that *DREAMTools* also include a plotting functionality with this challenge (see Figure 2).
**Scoring metric:** Spearman and Pearson correlations as well as AUROC and AUPR metrics are used, however, the Pearson correlation is used for the final ranking.

### D5C3: Systems Genetics challenges

**Description:** In this challenge, participants were asked to predict disease phenotypes and infer gene networks from Systems Genetics data. A first sub-challenge (SysGenA) made of simulated data considered 3 independent network sizes (100, 300 are 999), with 5 networks for each size. A second sub-challenge (SysGenB) provided training sets including phenotype, genotype, and gene expression data. Predictions of two phenotypes were required for 3 independent cases based on (1) only genotype data, (2) only gene expression data, and (3) both genotype and gene expression data ^23^.
**Scoring metric:** In the SysGenA sub-challenge, the final score is a function of AUPR and AUROC metrics (see D3C4 for details). In SysGenB, two phenotypes are scored using Spearman’s rank correlation. Their p-values are computed and the final score is then:
  $$S_{B}=-\left(\log p_{\text{pheno}1}+\log p_{\text{pheno}2}\right).\;(9)$$

### D5C4: Network Inference Challenge

**Description:** The goal of this challenge was to reverse engineer gene regulatory networks from gene expression data sets in *E. coli, S. cerevisiae, S. aureus*, and an *in silico* compendium. Each compendium is made of an expression matrix of *g* genes by *c* chip measurements. A set of decoy genes (about 5% of the compendium) were introduced by randomly selecting gene expression values from the compendium itself. The software GeneNetWeaver ^24^ was used to create the gene expression profiles for the *in silico* network.
**Scoring metric:** The final score is a function of AUPR and AUROC metrics (see D3C4 for details).

### DREAM6

### D6C1: Alternative Splicing

**Description:** RNA-splicing is the process of combining different exons of one gene towards the production of mature mRNA transcripts. Alternative splicing consists of assembling different combinations of exons; it plays an important role in transcriptome diversity including mammals. Shuffling of exons makes it possible for the same gene to code for different proteins. Besides, correct splicing is important for cells to function correctly. The challenge consisted of using short read RNA-seq data from Mandrill and Rhinoceros fibroblasts (about 100 nucleotides) so as to predict as many transcript isoforms as possible (generated by alternative splicing). The gold standard was created using selected target transcripts with read lengths between 1Kb and 2Kb nucleotides.
**Scoring metric:** Predictions are evaluated using the AUPR curve using a global alignment strategy: (1) precision at depth *i* in the prediction list was obtained by dividing by *i* the number of predicted transcripts in the first *i* predictions to which at least a gold standard transcript could be matched with a coverage and an identity of 95% or more. (2) Recall at depth *i* in the predicted list is calculated by dividing the number of gold standard transcripts that could be matched to the first *i* predicted transcripts with a coverage and an identity of 95% by the total number of transcripts in the gold standard.
  The AUPR values are computed for hESC (human embryonic stem cells) and Rhino IPSC (induced pluri-potent stem cells). The final score is the sum of the two AUPRs.

### D6C2: Parameter Estimation

This challenge was about the inference of the kinetic parameters of three gene regulatory networks using iterative optimization and a virtual experimental design ^25^. The challenge was proposed again in DREAM7 (see D7C2 section for details).

### D6C3: Expression Prediction

**Description:** The level by which genes are transcribed is largely determined by the DNA sequence upstream to the gene, known as the promoter region. The challenge consisted of predicting the promoter activity derived by a ribosomal protein (RP) promoter sequence. Participants were given a training set (90 RP promoters) for which both the promoter sequence and their activities are known and a test set (53 promoters) for which only the promoter sequence is known. The goal was to predict the promoter activity of the promoters in the test set ^26^.
**Scoring metric:** Four metrics are used ^26^: two distances between measured and predicted values and two differences in rank between measured and predicted values. The distances are based on a Pearson metric and a chi-square metric. The rank differences are based on the Spearman’s rank correlation and rank-square metric. Those 4 metrics have p-values denoted *p _j_* with *j* = 1..4 derived from null distributions based on participants’ submissions. The overall score is then:
  $$S=-\frac{1}{4}\prod_{j=1}^{4}p_{j}.\;(10)$$

### D6C4: FlowCAP2 Molecular Classification of Acute Myeloid Leukaemia Challenge

**Description:** Flow cytometry (FCM) has been widely used by immunologists and cancer biologists in the last decades as a biomedical research tool to distinguish different cell types in mixed populations, based on the expression of cellular markers. The goal of this challenge was to diagnose Acute Myeloid Leukaemia (AML) from patient samples using FCM data. In particular, participants were asked to find homogeneous clusters of cells, which can be used to discriminate between AML positive patients and healthy donors ^1^.
**Scoring metric:** Four metrics are used: AUPR, Matthews correlation coefficient (see Supplementary material), Jaccard similarity coefficient (size of the intersection divided by the size of the union of two sample sets), and Pearson correlation. The final score is the average of those four metrics and ranking amongst top performers is based on the Pearson correlation.

### DREAM7

### D7C1: Parameter Estimation

**Description:** Accurate estimation of parameters of biochemical models is required to characterize the dynamics of molecular processes. Consequently, effective experimental strategies for parameter identification and for distinguishing among alternative network topologies are essential. In this challenge, we created an *in silico* test framework under which participants could probe a network with hidden parameters. In addition, a virtual budget was provided to participants to buy experimental data (generated *in silico* with the model) mimicking the features of different common experimental techniques (e.g., microarrays and fluorescence microscopy). In a first sub-challenge, the topology and underlying biochemical structure of a 9-gene regulatory network was provided. Participants were asked to (i) estimate the 18 parameters of the model and (ii) predict outcomes of perturbations (time courses). The second sub-challenge provided an 11-gene regulatory network with 3 missing regulatory links to be guessed ^25^.
**Scoring metric:** For the first sub-challenge, two distances are computed. First, a distance *D*_param_ that is the mean of the mismatch between estimated and true parameters on a log-scale:
  $$D_{\text{param}}=\frac{1}{N_{p}}{\sum_{i=1}^{N_{p}}\left[\log\left(\frac{X_{i}^{p}}{X_{i}^{m}}\right)\right]}^{2},\;(11)$$
  with the number of parameters *N _*p*_* = 45. Second, a distance *D*_time course_ that is similar to Equation 1 (square errors):
  $$D_{\text{time course}}=\frac{1}{90}\sum_{k=1}^{3}\sum_{i=11}^{40}\frac{{\left[X_{k,i}^{p}-{\hat{X}}_{k,i}^{m}\right]}^{2}}{\sigma_{b}^{2}+\sigma_{s}^{2}{\left[X_{k,i}^{m}\right]}^{2}},\;(12)$$
  where *k* is a time course index, *i* a time index and the parameters *σ _*b*_* and *σ _*s*_* are set to 0.1 and 0.2, respectively (see D3C2 challenge for details). Since the initial time point was provided, the first 10 data points are ignored. From the participants’ submission a null distribution and p-values are computed and the final score is:
  $$S1=-log\left(p_{\text{param}}\times p_{\text{time course}}\right).\;(13)$$
  In the network topology sub-challenge, an ad-hoc distance based on the link and nature of the 3 missing regulations is used ^25^. Again, from the participant’s submission a null distribution and p-value is computed. The final score is then:
  $$S2=-log\left(p_{\text{topology}}\right).\;(14)$$

### D7C2: Breast Cancer Prognosis

**Description:** In the breast cancer prognosis challenge, the goal was to assess the accuracy of computational models designed to predict breast cancer survival. Participants were asked to build computational models based on clinical information about the patient’s tumor. In addition, genome-wide molecular profiling data including gene expression and copy number profiles were provided ^9^.
**Scoring metric:** Models were scored by calculating the *exact concordance index* between the predicted survival and the true survival information in the validation data set (accounting for the censor variable indicating whether the patient was alive at last follow-up). See Supplementary material for details.

### D7C3: The DREAM Phil Bowen ALS Prediction Prize4Life

**Description:** ALS is a fatal neurodegenerative disease. One important obstacle to understanding and developing an effective treatment for ALS is the heterogeneity of the disease course, ranging from under a year to over 10 years. The more heterogeneous the disease, the more difficult it is to predict how a given patient’s disease will progress. ALS status is defined by a functional scale called ALS Functional Rating Scale (ALSFRS). ALS progression between two time points can be defined as the slope between two ALSFRS values. The goal of the challenge was to predict the future progression of disease in ALS patients based on the patient’s current disease status and data (e.g., family history data, vital signs, lab data...) ^8^.
**Scoring metric:** Two ALSFRS values are available for each patient, providing the actual slope *X* across patients. The accuracy of predicted slopes X^ from participants is assessed using the root mean square error.

### D7C4: NCI-DREAM Drug Sensitivity and Synergy Prediction

**Description:** The connection between molecular measurements and cellular drug response is central to precision medicine. Two sub-challenges were run to evaluate methods that leveraged -omics measurements to predict drug response in human cell lines. The first sub-challenge was to predict drug sensitivity in breast cancer cell lines by integrating multiple –omics data types ^27^. The second sub-challenge was to predict drug synergy/antagonism in a B cell lymphoma cell using gene expression and copy number alterations ^28^.
**Scoring metric:** In sub-challenge 1, teams were asked to predict the rank order of cell lines treated with 28 drugs. An aggregate scoring method was developed that we called the weighted, probabilistic concordance-index ( *wpc*-index), a variant of the concordance index (see Section 1.2.5 for details). Indeed, drug measurements vary across experiments and gold standard ranked list of cell lines by drugs is subject to noise. The pooled variance was calculated and taken into account when scoring and the final *wpc*-index was the weighted average over all drugs. Statistical significance was calculated by comparing a team’s *wpc*-index to the empirical null distribution of random sets of predictions. False Discovery Rates (FDRs) were calculated to account for the multiple testing hypotheses given by the number of teams that submitted predictions to the challenge. Teams were also scored according to a *resampled* Spearman correlation. Full details of the scoring methodology can be found in the Supplementary Note 3 in Costello, *et al.* ^27^.
  In sub-challenge 2, teams were asked to predict the rank order of drug combinations for 14 drugs from the most synergistic to most antagonistic. For each drug combination, drug response was measured on the Ly3 cell line and Excess over Bliss (EoB) was calculated as the average over five replicates *e _i_* with the corresponding standard deviation *s _i_*. The definition of Bliss additivisim (or Bliss independence) can be found in Borisy *et al.* ^29^. Similar to sub-challenge 1, the scoring method was a modification of the concordance index, taking into account the probabilistic nature of the EoB calculations. A leave-one-out approach (leave-one-drug-out) was used for p-value estimation and FDR correction was applied. Additionally, the resampled Spearman scoring approach was used as a second scoring method. Full details of the scoring methodology can be found in Supplementary Note 1 in Bansal *et al.* ^28^.

### DREAM8

### D8C1: HPN-DREAM Breast Cancer Network Inference

**Description:** This challenge aimed to advance and assess our ability to infer causal protein signaling networks and predict protein time-courses in a complex, mammalian setting. Participants were provided with protein time-course data from four breast cancer cell lines under various ligand stimuli and inhibitor perturbations. The challenge consisted of three sub-challenges. Sub-challenge 1 tasked teams with inferring causal signaling networks specific to each of 32 contexts defined by combination of cell line and stimulus. In contrast to networks that simply describe correlations between nodes, a directed edge in a causal network predicts that an intervention on the parent node will lead to a change in abundance of the child node. For sub-challenge 2, teams were asked to predict context-specific phosphoprotein time-courses under an unseen inhibitor perturbation. Sub-challenges 1 and 2 also consisted of companion tasks based on *in silico* data. Sub-challenge 3 (not part of *DREAMTools*) asked teams to devise novel ways to visualize these data. A full description of the challenge can be found in 10.
**Scoring metric:** For sub-challenge 1, since there were no gold standard causal networks for the experimental data task, a scoring procedure was developed that used held-out interventional test data to assess the causal validity of submitted networks. In brief (for full details see 10), the held-out test data consisted of time-courses for the same 32 contexts, but obtained under an inhibitor not contained in the training data (an mTOR inhibitor). The test data were used to identify, for each context, proteins that show salient changes in abundance under mTOR inhibition (relative to baseline). This provides a ’gold standard’ set of descendants of mTOR for each context and these were compared against descendants of mTOR in submitted networks, resulting in 32 AUROC scores for each team. Teams were ranked within each context and the final score was the mean rank across the 32 contexts. For the *in silico* data task, the gold standard (data-generating) causal network was known and could be directly compared against submissions to calculate AUROC scores. For sub-challenge 2 experimental data task, team predictions of protein time-courses under mTOR inhibition were directly compared against the held-out test data (also obtained under mTOR inhibition). Performance was assessed using root mean squared error (RMSE). Teams were ranked by RMSE within each (cell line, phosphoprotein) pair and the final score was the mean rank across all pairs. A similar procedure was used for the *in silico* data task.

### D8C2: NIEHS-NCATS-UNC DREAM Toxicogenetics Challenge

**Description:** The challenge was designed to investigate the predictability of cytotoxicity in a population in response to environmental compounds and drugs. *In vitro* cytotoxicity screening was performed for 884 lymphoblastoid cell lines perturbed with 156 compounds. Genotype and transcriptional data for the cell lines were available as part of the 1000 Genomes Project ( www.1000genomes.org) and structural attributes for the compounds were also provided. Participants were provided with training data consisting of the cytotoxic response for 620 cell lines and 106 compounds. Two sub-challenges were proposed: (1) prediction of individual cytotoxicity for 264 new individuals in response to the 106 compounds of the training set and (2) prediction at a population-level cytotoxicity (median and interquantile range) for 50 new compounds. Full description of the challenge is available in 7.
**Scoring metric:** Sub-challenge 1: for each submission, Pearson correlation and probabilistic concordance index (wpc) are computed for each of the 106 compounds in the test set across the 264 individuals. For each metric, teams are ranked separately for each compound and an average rank is then computed across compounds. The final rank is the average of the two intermediate ranks.
  Sub-challenge 2: for each submission, Pearson correlation and Spearman correlation are computed for the predicted median cytotoxicity and interquantile range across the 50 compounds in the test set. Submissions are ranked separately for each population parameter (i.e. median and interquantile range) and then the final rank is the average of the two intermediate ranks.

### D8C3: Whole-Cell Model Parameter Estimation Challenge

**Description:** Participants were challenged to estimate the parameters of a modified whole-cell model of a slow-growing mutant strain of the bacterium *Mycoplasma genitalium* ^30^. Participants were given eight types of simulated data generated using the mutant strain. Participants were also given credits to purchase additional perturbation data generated by modifying the values of individual parameters of the mutant strain. Full description of the challenge is available in 31.
**Scoring metric:** As in the D7C1 challenge (See D7C1 section), submissions were scored based on a combination of their parameter and prediction distances ( Equation 13). The parameter distance was computed as the average log ratio of the estimated and true parameter values ( Equation 11). The prediction distance was computed as the average Euclidean distance between the estimated and true *in silico* phenotypes, scaled by their variances. This scoring function is not included in *DREAMTools*. This scoring function is implemented in MATLAB, and is available open-source at GitHub ( https://github.com/CovertLab/wholecell). A complete working example of this scoring function, including the gold standard, is available at Synapse ( https://www.synapse.org/#!Synapse:syn1876068/wiki/232963).

### DREAM8.5

### D8dot5C1: Rheumatoid Arthritis Responder

**Description:** The goal of this challenge was to use a crowd-based competition framework to develop a validated molecular predictor of anti-TNF response in Rheumatoid Arthritis (RA). We used the whole genome SNP data derived from two cohorts: 2,706 anti-TNF treated RA patients combined across 13 collections of European ancestry ^32^, and 591 patients in the CORRONA CERTAIN study ^33^. Treatment efficacy was measured using the absolute change in disease activity score in 28 joints ^34^ (DAS28) following 3–6 months of anti-TNF treatment. The challenge was devised into two components. Sub-challenge 1: predict treatment response as measured by the change in disease activity score (DAS28) in response to anti-TNF therapy. Sub-Challenge 2: identify poor responders as defined by EULAR ^35^ criteria for non-response (20% of the study population).
**Scoring metric:** In sub-challenge 1, each participant submission is scored independently using the Spearman correlation. In sub-challenge 2, each submission is scored independently using the AUPR and AUROC metrics (same as D2C3).

### DREAM9

### D9C1: The Broad-DREAM Gene Essentiality Prediction Challenge

**Description:** Essential genes are those genes of an organism that are thought to be critical for its survival. In this challenge, participants were given a set of training gene dependency/essentiality scores from a set of cancer cell lines with expression data, copy number data, and mutation data. The goal was to develop predictive models that can infer gene dependencies/essentialities using the provided molecular features. Three sub-challenges included (i) building a model that predicts all gene essentiality scores in a held-out test set, using any feature data, (ii) predicting a subset of gene essentiality scores using only *N* = 10 gene expression, copy number, or mutation features per gene and (iii) same as sub-challenge 2 with *N* = 100. For sub-challenges 2 and 3, a smaller list of *prioritised* 2647 genes was selected considering profiles of the gene essentiality data, cancer related genes and evidence of the gene to be a potential drug target.
**Scoring metric:** For all sub-challenges, prediction performances are assessed in terms of Spearman’s rank correlation coefficient. We first calculate the Spearman’s rank correlation coefficient for each gene between the measured and predicted gene-level scores over held-out cell lines. For each submission, the overall score is calculated as the average correlation over all genes (all genes for sub-challenge 1 and all prioritized genes for sub-challenges 2 and 3).

### D9C2: AML Outcome Prediction

**Description:** AML is a cancer of the bone marrow and the blood. Mutations in the myeloid line of blood stem cells lead to the formation of aberrant myeloid blasts and white blood cells. If untreated, these highly proliferative cancerous cells impede the development of normal blood cells and eventually cause death. In this challenge, participants had to predict the outcome of treatment of AML patients (resistant or remission) as well as their remission duration and overall survival based on clinical cytogenetics, known genetics markers and phosphoproteomic data. Three sub-challenges were conducted. In the first, participants were asked to predict which AML patients will have complete remission or will be primary resistant. In sub-challenge 2, participants were asked to predict remission duration for patients who have complete remission.
**Scoring metric:** In sub-challenge 1, the scoring methods are the AUROC and balanced accuracy (BAC), defined in Section 1.1. In sub-challenge 2 and 3, the scoring methods are the concordance index ( CI) and Pearson correlation coefficient (see Section 1.2.5). The Pearson correlation coefficient is used to measure correlation between predictions of remission duration and actual remission duration. In those sub-challenges, the final rank is the average of the two intermediate ranks.

### D9C3: Alzheimer’s Disease Big Data

**Description:** The goal of the Alzheimer’s Disease (AD) challenge was to identify accurate predictive biomarkers that can be used to improve AD diagnosis and treatment. In order to build predictive models, participants were given genetics and brain imaging data in combination with cognitive assessments, biomarkers and demographic information from cohorts ranging from Cognitively Normal (CN) to Mild Cognitively Impaired (MCI) to individuals with Alzheimer’s Disease (AD). An essential metric for diagnosis is the Mini-mental state examination (MMSE) score at baseline and at the 24 month follow-up visit. Three sub-challenges were conducted to (i) predict the change in cognitive scores 24 months after initial assessment (ii) predict the set of cognitively normal individuals whose biomarkers are suggestive of amyloid perturbation and (iii) classify individuals into diagnosis groups using MR imaging.
**Scoring metric:** In the first sub-challenge, participants were asked to predict the change in cognitive scores using (i) clinical covariate only or (ii) clinical covariate and additional genetics variables. Those two predictions are scored using Pearson and Spearman correlations leading to 4 ranks across submissions, which are average to provide the final rank.
  In the second sub-challenge, the problem was to understand how some people maintain normal cognitive function in the presence of amyloid pathology. The set of cognitively normal individuals predicted by participants includes the ranking of these subjects (from the most discordant to the least discordant), the confidence in the ranking, and if the subject is discordant or concordant. The final score is the average of the AUROC and BAC values.
  In the third sub-challenge, participants were asked to classify individuals to differentiate AD patients from others using MR imaging using the MMSE as a confidence score. Two scores are computed to rank the submissions based on (1) the Pearson correlation of the predicted MMSE with the measured MMSE scores and (2) the concordance correlation coefficient (CCC) (see Section 1.2.4) for agreement on a continuous measure between observed and predicted MMSE. Again, final ranking is the average of these two ranks. Note that the percentage of correctly classified subjects in each of the three diagnostic classes is used to resolve ties.

### D9C4: ICGC-TCGA-DREAM Somatic Mutation Calling Challenge

**Description:** The detection of somatic mutations from cancer genome sequences is key to understanding the genetic basis of disease progression, patient survival and response to therapy. The goal of the somatic mutation calling (SMC) challenge is to identify the most accurate mutation detection algorithms using as input whole-genome sequencing (WGS) data from tumor (prostate and pancreatic) and normal samples ^36^.
  There were two sub-challenges called *Intel-10 SNV* and *ITM1-10 SV*. Single nucleotide variants (SNVs) are alterations of a single base within the DNA code, and often cause sensitivity to specific drugs. A typical cancer may contain tens of thousands of SNVs. Structural variations (SVs) are duplications, deletions or rearrangements of large segments of the genome and are often described as being the primary cause of cancer.
**Scoring metric:** Genomic variant detectors are classifiers. The performance of the predictive algorithms from the participating challenge teams are ranked using the validation data to compute the sensitivity, specificity and balanced accuracy.

### DREAM9.5

### D9dot5C1: DREAM Olfaction Prediction Challenge

**Description:** The goal of this challenge was to predict how a molecule smells from its physical and chemical features. We provided a large unpublished data set based on extensive smell-testing of 49 human subjects asked to sniff 476 different odor chemicals. Subjects were asked to tell us how pleasant the odor is, how strong the odor is, and how well the smell percept matches a list of 19 descriptors. To complement these perceptual data, we provided physical-chemical information about each odor molecule. Two sub-challenges were proposed. In the first, participants had to predict individual odor intensity, odor valence (pleasantness/unpleasantness) and the matrix of 19 odor descriptors (at high intensity) for each of the 49 subjects. In the second sub-challenge, the mean and standard deviation of the odor intensity, odor valence (pleasantness) and matrix of 19 odor descriptors (at high intensity) were requested.
**Scoring metric:** Out of the 476 odor chemicals, 338 were provided as a training set and 69 were used as a test set for the final scoring. In sub-challenge 1, the Pearson correlation across the 69 odors for intensity (int) and pleasantness/valence (ple) are computed and denoted *r*_int_ and *r*_ple_, respectively. The mean for all 49 individuals is computed and denoted ${\bar{r}}_{\mathrm{int}}$ and ${\bar{r}}_{\text{ple}}$. Similarly, for the descriptors, the Pearson coefficient for each of the 69 odor is averaged across the individuals and descriptors and denoted ${\bar{r}}_{\text{dec}}$. The z-scores are calculated by subtracting the average Pearson correlations and scaling by the standard deviation of a distribution based on a randomization of the gold standard. The final score is the average of the z-scores.
  For sub-challenge 2: Instead of using the mean (across 49 individuals) of the Pearson correlation (across the 69 odors), the Pearson correlation of the mean intensity and standard deviation (across 49 individuals) was used. This leads to 6 values (2 for intensity, 2 for valence and 2 for descriptors). Again, z-scores are calculated from an empirical null distributions and the final score is the average of the z-scores.

### D9dot5C2: Prostate Cancer DREAM Challenge

**Description:** This challenge focused on predicting survival using patients’ clinical variables with the goal to improve prognostic models and toxicity of docetaxel treatment in patients with metastatic castrate resistant prostate cancer (mCRPC). Over 100 clinical variables were summarized across four phase III clinical trials with over 2,000 mCRPC patients treated with first-line docetaxel. There were two sub-challenges. Sub-challenge 1a was to predict overall patient survival and sub-challenge 1b was to predict the exact time to event for each patient. Sub-challenge 2 was to predict if a patient will be discontinued from docetaxel treatment because of adverse events. The primary benefit of this Challenge will be to establish new quantitative benchmarks for prognostic modeling in mCRPC, with a potential impact for clinical decision making and ultimately understanding the mechanism of disease progression.
**Scoring metric:** Participants were asked to produce “risk scores” for each patient for sub-challenge 1a and the exact time to death for sub-challenge 1b. There were two metrics used to score participants for sub-challenge 1a, namely the integrated AUC (iAUC) as defined in the timeROC package in R and the concordance index (see Section 1.2.5). Sub-challenge 1b was scored using the root mean squared error (RMSE).
  For sub-challenge 2, participants were asked to supply a “risk score” and a discrete variable equal to 1 if the patient is predicted to discontinue within 3 months and 0 otherwise. Submissions were scored using the AUPR metric as defined in the ROCR package in R.

### DREAM10

### D10C1: ALS Stratification Prize4Life

**Description:** This challenge is a follow-up on to the DREAM 7 ALS Prize 4 Life Challenge (see D7C3 for details). It focuses on predicting the progression and survival of ALS patients. One objective of the challenge is to leverage the PRO-ACT database of more than 8,000 cases as the challenge training set. The challenge will include several unpublished data sets to be used for model validation.
**Scoring metric:** This is an on-going challenge. The scoring metric will be based on concordance index, Pearson correlation and root-mean-squared deviation.

### D10C2: ICGC-TCGA DREAM Somatic Mutation Calling Tumor Heterogeneity (SMC-Het)

**Description:** This challenge is a follow-up on to D9C4 challenge (somatic mutation calling). This challenge’s focus is to identify the best subclonal reconstruction algorithms and to identify the conditions that affect their performance. See Section D9C4 for details.
**Scoring metric:** This is an on-going challenge. The scoring metric has not been released yet (September 2015).

### D10C3: ICGC-TCGA DREAM Somatic Mutation Calling RNA (SMC-RNA)

**Description:** This challenge is a follow-up on to D9C4 challenge (somatic mutation calling). See Section D9C4 for details.
**Scoring metric:** This is an on-going challenge. The scoring metric has not been released yet (September 2015).

## Conclusions

The organization of a collaborative competition such as the DREAM challenges is a complex task that starts by identifying a currently important and unresolved scientific problem, acquiring relevant data sets, engaging a community of participants, and implementing an appropriate scoring methodology. Participants can submit their solutions (e.g., predictive models or predictions) which are then scored and ranked, and the results are shown on a public leaderboard. Once the challenge is closed, those leaderboards can be used as a benchmark for further development of methods. To promote scientific reproducibility as well as post-challenge use, DREAM provides via Sage Bionetwork’s Synapse platform the resources to help researchers access data and leaderboards of previous challenges.

In this paper, we presented *DREAMTools* to provide a uniform framework where researchers can easily assess and compare new methods against benchmarks. *DREAMTools* gathers most of the scoring functions used in previous DREAM challenges. *DREAMTools* uses Python as a glue language known for its flexibility and ability to call other languages. Currently, about 80% of the closed challenges are available. The remaining challenges are either in the process of being included or hosted on external websites. Future versions of *DREAMTools* will aim at making available as many closed challenges as possible including newly closed challenges.

*DREAMTools* will help researchers who wish to test their algorithms against existing benchmarks. Indeed, templates can be downloaded and used to create predictions, which can then be tested. The gold standards are also available together with the relevant scoring functions. Since *DREAMTools* makes use of an object oriented approach, it will ease the integration of future challenges thereby facilitating scoring in upcoming challenges. *DREAMTools* can also be used as a place to retrieve metadata and information about a challenge. *DREAMTools* can be used as a standalone application or as a library making it a useful tools to be included in other software or pipelines. Developers who use the proposed layout will not need to change anything regarding the standalone application that will automatically recognize the challenge. In summary, we hope that *DREAMTools* will be a useful tool for researchers interested in benchmarking their methods against the state-of-the-art as defined by previous DREAM challenges, and to those developing new collaborative competitions within DREAM or elsewhere.

## Software availability

### Software releases are available from the Pypi website

https://pypi.python.org/pypi/dreamtools

### Latest source code is hosted on GitHub website

http://github.com/dreamtools/dreamtools

### Archived source code at the time of publication

http://www.dx.doi.org/10.5281/zenodo.47949 ^37^

### Bug report and feature requests

https://github.com/dreamtools/dreamtools/issues

### Documentation

http://dreamtools.readthedocs.org

### License

BSD 3-clause license (“BSD NEW” or “BSD Simplified”)

## Figures and Tables

**Table 2.** Contingency table for a binary classifier.

		Condition/Gold standard	
		Condition Positive (P)	Condition Negative (N)
Prediction	Positive	**T**rue **P**ositive (TP)	**F**alse **P**ositive (FP)
	Negative	**F**alse **N**egative (FN)	**T**rue **N**egative (TN)

## Supplementary material

This section covers tools used in the DREAM scoring functions.

### 1.1 Binary classification problem

Binary classification is the task of classifying the elements of a data set into two groups (e.g., a medical testing to determine if a patient has certain disease or not). It has been used in many of the DREAM challenges ^16^ to evaluate prediction performance as compared to a gold standard. Given a binary classifier, there are four possible outcomes that can be arranged in a 2 × 2 contingency table filled with true positives ( *TP*), true negatives ( *TN*), false positives ( *FP*), and false negatives ( *FN*). False positives are also known as false alarms or type I error and the false negative are also known as miss or type II error. The contingency table generally fills columns with actual values and rows with the prediction. True positive being in the top left corner. See Table 2.

From the contingency table, many metrics can be used to measure the performance of a classifier. Some may be more appropriate than others depending on the problem posed or the prevalence of the classes considered (see below). A useful technique to visualize and select classifiers is the Receiver Operating Characteristics (ROC) graph, which have long been used in signal detection theory ^38^. Efficient algorithms to compute ROC graphs and practical issues can be found in 39. The next section will describe the ROC analysis in details. Before, let us provide some common equations and terminology used in the evaluation of binary classifiers and ROC analysis.

The prevalence mentioned earlier (also known as balance) is the ratio of positive conditions to the total population:

$$\text{prevalence}=\frac{P}{P+N}\;(15)$$

where *P* is the total number of positives (i.e., *TP* + *FN*) and *N* the total number of negatives (i.e., *TN* + *FP*). The total population is denoted *T* = *P* + *N*.

From the 4 basic numbers of the contingency table, 8 ratios can be obtained by dividing those numbers by either the sum of the rows or the sum of the columns.

The column ratios are the proportion of the population with a given condition (positive or negative).

1. The true positive rate (TPR) is the ratio of true positive by the sum of positive conditions. The TPR value also known as **sensitivity** or **recall** is a measure of completeness:
  $$TPR=\text{recall}=\text{sensitivity}=\frac{TP}{P}\;(16)$$
2. The false negative rate (FNR) also known as miss rate is:
  $$FNR=\text{miss rate}=\frac{FN}{P}\;(17)$$
3. The false positive rate (FPR):
  $$FPR=\text{fall out}=\frac{FP}{N}\;(18)$$
4. The true negative rate (TNR) also known as **specificity:**
  $$TNR=\text{specificity}=\frac{TN}{N}\;(19)$$

Note that these ratios are independent of the total number of conditions (i.e., there are independent of the prevalence).

The row ratios are computed as follows:

1. The positive predictive values (PPV) or **precision** is a measure of fidelity:
  $$PPV=\text{precision}=\frac{TP}{TP+FP}\;(20)$$
2. The false discovery rate (FDR):
  $$FDR=\frac{FP}{TP+FP}\;(21)$$
3. The false omission rate (FOR):
  $$FOR=\frac{FN}{FN+TN}\;(22)$$
4. The negative predictive values (NPV):
  $$NPV=\frac{TN}{FN+TN}\;(23)$$

Note that these ratios have denominator that combines positive and negative (i.e. they depends on the prevalence).

There are a number of other metrics derived from those 8 numbers. We can mention the *accuracy* and *F-measure*. The accuracy measures the fraction of all instances that are correctly categorized:

$$ACC=\frac{TP+TN}{P+N}.\;(24)$$

Another related measure used for instance in D9C2 challenge (See Section D9C2) is balanced accuracy (BAC):

$$BAC=\frac{1}{2}\left(\frac{TP}{P}+\frac{TN}{N}\right)\;(25)$$

in other words, the arithmetic average of the sensitivity and specificity. The BAC metric avoids inflated performance estimates on imbalanced data sets.

The F-measure or balanced F-score (F1 score) is the harmonic mean of precision and recall:

$$\text{F-measure}={\text{F}}_{1}=2\frac{\text{precision}\times \text{recall}}{\text{precision}+\text{recall}}\;(26)$$

The F-measure is often used in the field of information retrieval for document classification with large scale data where performance needs to place more emphasis on either precision or recall. Note, however, that the F-measure does not take the true negatives into account, which appears clearly in this other formulation of the F-measure/F1 score:

$$\text{F-measure}={\text{F}}_{1}=\frac{2TP}{2TP+FP+FN}\;(27)$$

***1.1.1 AUPR and AUROC curves.*** It is common to explore complementary metrics simultaneously varying a cutoff of the decision boundary. The pair precision-recall is used to estimate a first metric known as the precision-recall curve (AUPR). The true positive rate and false positive rate pair is used to estimate a second metric called AUROC (Area Under the receiver operating characteristic (ROC) curve).

The precision and recall are therefore functions of a varying parameter, *k*, in a precision-recall curve and can be expressed as:

$$precision(k)=\frac{TP(k)}{TP(k)+FP(k)}=\frac{TP(k)}{k}\;(28)$$

and:

$$recall(k)=\frac{TP(k)}{P}\;(29)$$

similarly, the receiver operating characteristic (ROC) curve explores the trade-off between true and false positive rates as a function of a varying *k* parameter. The TPR and FPR are denoted:

$$TPR(k)=\frac{TP(k)}{k}\;(30)$$

and:

$$FPR(k)=\frac{FP(k)}{N}\;(31)$$

The AUROC is a single measure that is the integral of the TPF/FPR curve. Similarly the AUPR is a single measure that is the integral of the recall-precision curve.

The values of recall and precision range from zero to one with one being the optimal value for precision and *min*( *k*/ *P*, 1) being the optimum for recall at depth *k*. The precision-recall curve explores changes in accuracy as *k* increases.

Note that in the case of a discrete classification, the ROC curves contains only 1 point.

***1.1.2 Matthews correlation coefficient.*** An additional measure that can easily be computed is the Matthews correlation coefficient (MCC), which is a measure of the quality of a binary classification ^40^. It can be used even if the classes are of very different sizes (low or high prevalence). The MCC can be calculated directly from the confusion matrix using the formula:

$$\text{MCC}=\frac{TP\times TN-FP\times FN}{\sqrt{\left(TP+FP\right)\left(TP+FN\right)\left(TN+FP\right)\left(TN+FN\right)}}\;(32)$$

The MCC is also known as the *phi coefficient*, which is the chi-square statistic for a 2 × 2 contingency table:

$$\text{MCC}=\sqrt{\frac{\chi^{2}}{T}}\;(33)$$

where *T* is the total number of observations.

### 1.2 Regression Problem

In a regression problem the task is to predict the numerical values for a set of variables called the dependent variables from a different set of variables (independent variables).

***1.2.1 Root Mean Square Error.*** The root-mean squared error (RMSE) that averages the quadratic errors of the individual measurements, that is, the differences between the actual values of the dependent variables ( *y*) and those predicted from the model ( *ŷ*).

$$RMSE=\;\sqrt{\frac{1}{n}\sum_{i=1}^{n}{\left(y_{i}–{\hat{y}}_{i}\right)}^{2}}\;(34)$$

***1.2.2 Pearson correlation.*** The Pearson product-moment correlation coefficient (PCC) is a measure of the linear correlation between two variables. The measure indicates a total positive correlation (+1), no correlation (0) or total negative correlation (-1). For a population, it is usually denoted *ρ _X,Y_* and defined as

$$\rho_{X,Y}=\frac{cov(X,Y)}{\sigma_{X}\sigma_{Y}}\;(35)$$

Where cov is the covariance between the two variables and σ the standard deviation. For a sample, we can substitute estimates of the covariances and variances based on the sample *x* and *y* of length *n*. One formulation for the sample Pearson correlation coefficient is:

$$r_{xy}=\frac{\sum x_{i}y_{i}–n\bar{x}\bar{y}}{\sqrt{\sum x^{2}–n{\bar{x}}^{2}}\sqrt{\sum y^{2}–n{\bar{y}}^{2}}}\;(36)$$

with $\bar{x}$ and $\bar{y}$ the sample means of *x* and *y*.

***1.2.3 Spearman’s rank correlation.*** Spearman’s rank correlation coefficient or Spearman’s rho denoted *ρ* is a nonparametric measure of statistical dependence between two variables. It assesses how well the relationship between two variables can be described using a monotonic function. Spearman’s coefficient is appropriate for continuous and discrete variables, including ordinal variables.

The Spearman correlation coefficient is defined as the Pearson correlation coefficient between the ranks of variables. For a sample of size *N*, the *N* raw scores *X _i_*, *Y _i_* are converted to ranks *x _i_*, *y _i_*, and *ρ* is computed from:

$$\rho =1-\frac{6\sum d_{i}^{2}}{N(N^{2}-1)}.\;(37)$$

where *d _i_* = *x _i_* – *y _i_*, is the difference between ranks. Note that identical values are assigned a rank equal to the average of their positions in the ascending order of the values.

***1.2.4 Concordance correlation coefficient.*** The concordance correlation coefficient (CCC) measures the agreement between two variables ^41^ and is denoted *ρ _c_*:

$$\rho_{c}=\frac{2\rho \sigma_{x}\sigma_{y}}{\sigma_{x}^{2}+\sigma_{y}^{2}+{\left(\mu_{x}-\mu_{y}\right)}^{2}}\;(38)$$

It can be used as a measure of the correlation between two variables around the 45 degree line from the origin. It is used for instance in challenge D9C3 ^41^.

***1.2.5 Concordance index.*** In D7C2 challenge (See Section D7C2), an exact concordance index was used as a scoring metric. The concordance index (c-index) was first introduced to the biomedical community in 42. It is a measure of association between the predicted and observed failures in case of right censored data. In the absence of censored data, the c-index estimates the Mann–Whitney parameter. Note that censoring is a condition in which the value of a measurement or observation is only partially known (e.g., impact of a drug on mortality rate for living subjects that may die before the end of the study).

We use here below the same formulation as in the Supplementary Note 3 in Costello, *et al.* ^27^. For a given drug *d*, we define *R _d_* = { *r*_1_, *r*_2_,..., *r _N_*} a rank order for *N* predictions (e.g., *N* cell lines). Similarly, we define *G _d_* = { *g*_1_, *g*_2_,..., *g _N_*} a rank order for the gold standard.

$$\text{c}-\text{index}=c(G_{d},R_{d})=\frac{2}{N(N-1)}\sum_{i<j}h(g_{i},g_{j},r_{i},r_{j})\;(39)$$

where

$$h(g_{i},g_{j},r_{i},r_{j})=\left\{ \begin{array}{l} 1,\;\text{ if }(g_{i}>g_{j}\&r_{i}<r_{j})\vee (g_{i}<g_{j}\&r_{i}>r_{j})\\ 0.5,\text{ if }(g_{i}=g_{j})\\ 0,\;\text{ if }(g_{i}>g_{j}\&r_{i}>r_{j})\vee (g_{i}<g_{j}\&r_{i}<r_{j}) \end{array}\right.\;(40)$$

In the previous formulation of the c-index, the variance of the gold standard data set is not taken into account. A probabilistic c-index (denoted pc-index) was introduced in Costello *et al.* ^27^, Bansal *et al.* ^28^ and calculated as follows:

$$\begin{array}{l} \begin{array}{l} \text{pc}-\text{index}=pc(G_{d},R_{d},s_{d}^{2})\\ \;=\frac{2}{N(N-1)}\sum_{i<j}h(g_{i},g_{j},r_{i},r_{j},\sqrt{s_{d}^{2}}) \end{array}\;(41) \end{array}$$

where

$$h(g_{i},g_{j},r_{i},r_{j},s_{d})=\left\{ \begin{array}{l} \frac{1}{2}\left(1+er\;f\left(\frac{g_{i}-g_{j}}{2s_{d}}\right)\right),\text{ if }({\text{r}}_{\text{i}}<{\text{r}}_{\text{j}})\\ 0.5,\;\;\;\;\;\;\;\;\;\;\text{ if }(g_{i}=g_{j})\\ \frac{1}{2}\left(1+er\;f\left(\frac{g_{j}-g_{i}}{2s_{d}}\right)\right),\text{ if }(r_{i}>r_{j}) \end{array}\right.\;(42)$$

and $s_{d}^{2}$ is the pooled variance to account for the uncertainties of the gold standard. The equation *erf* is the standard error function $erf(a)=\frac{2}{\sqrt{\pi }}\int_{0}^{a}e^{-t^{2}}dt.$

In the case of the NCI-DREAM challenges, the final score was a weighted average of the *pc*-index, which we termed the weighted, probabilistic c-index ( *wpc*-index), where the weights *w _d_* for each drug *d* reflect the quality of the measured data for *d*, accounting for the range of total response and missing values. The *wpc*-index is calculated as:

$$wpc-\text{index}=\frac{\sum_{d}w_{d}\cdot pc_{d}}{\sum_{d}w_{d}}\;(43)$$
