# Supplementary material for: DREAMTools: a Python package for scoring collaborative challenges
Source: F1000Res. 2016 Apr 8;4:1030. Originally published 2015 Oct 9. [Version 2] doi: 10.12688/f1000research.7118.2 (PMC4837986; doi:10.12688/f1000research.7118.2)
Supplement: Peer review history

# Peer review: Referee response for version 2

**DOI:** 10.5256/f1000research.9054.r13274

**Published:** 2016-04-18

**Authors:** Konrad Hinsen

**Affiliations:** 1 Centre de Biophysique Moléculaire (UPR4301 CNRS), Rue Charles Sadron, Orléans, France

The revision addresses the technical issue that prevented a full evaluation of the software being described. The solution chosen by the authors, which is making their software available through bioconda in addition to publication of the source code on GitHub, is entirely satisfactory both for me as a reviewer and for potential users of the software.

I have read this submission. I believe that I have an appropriate level of expertise to confirm that it is of an acceptable scientific standard.

# Peer review: Referee response for version 1

**DOI:** 10.5256/f1000research.7664.r11442

**Published:** 2015-12-23

**Authors:** Nicola Mulder

**Affiliations:** 1 Institute of Infectious Disease and Molecular Medicine, University of Cape Town, Cape Town, South Africa

This paper describes DREAMTools, which is a package that enables scoring of submissions for DREAM challenges. The paper is well written and this is certainly a useful tool. Benchmarking is essential in all aspects of tool or algorithm development, and the package described here will be helpful for evaluating the challenges. I did find the paper a bit too long. While it is interesting reading about the different examples DREAM challenges, these have been available on the DREAM challenges website, so perhaps a few representative examples could be selected from all those presented in the paper to illustrate the point.

Like the other reviewer, my only reservation is the difficulty in getting the package installed, and though work has been done to make it compatible with Python 3, perhaps some troubleshooting tips would help users to overcome dependency issues.

I have read this submission. I believe that I have an appropriate level of expertise to confirm that it is of an acceptable scientific standard, however I have significant reservations, as outlined above.

# Response

**Published:** 2016-01-18

**Authors:** Thomas Cokelaer

**Affiliations:** EMBL European Bioinformatics Institute, UK

Dear Nicola,

Thanks a lot for your review and comments.

It is true that more challenges will be added in the future and that the paper would be become even longer ! We are thinking about reducing the paper's length in future versions and representative examples would be a good solution.

As for the installation issues that other reviewers' faced, we have improved the code  and documentation to take into account any issues and will keep improving the installation. As mentionned in other reports's comments, we will provide a DREAMTools pre-compiled package in the close future that should help even more.

Thanks again.

Thomas Cokelaer on behalf of DREAMTools developers.

# Response

**Published:** 2016-03-30

**Authors:** Thomas Cokelaer

**Affiliations:** EMBL European Bioinformatics Institute, UK

Dear Nicola,

For your information, DREAMTools is now available on the bioconda (https://bioconda.github.io/) channel of Anaconda (https://www.continuum.io/downloads). Installation of the latest DREAMTools release should now be easier for MAC and Linux users as explained within the online documentation (http://dreamtools.readthedocs.org/en/latest/#installation).

Best

Thomas

# Peer review: Referee response for version 1

**DOI:** 10.5256/f1000research.7664.r11489

**Published:** 2015-12-21

**Authors:** Rafael Najmanovich

**Affiliations:** 1 Department of Biochemistry, University of Sherbrooke, Sherbrooke, QC, Canada

The authors set out to create a framework to easily access data from a large number past DREAM challenges and assess new predictions for these using appropriate the evaluation metrics defined in the respective challenges. My main reservation is that I find that the DREAMTools package is, contrary to what the authors claim, of little use at the moment. Perhaps in a future where the official DREAM challenges make currently open challenges available via DREAMTools, this will be more useful. At the moment, any group interested in benchmarking their prediction method for a particular challenge can easily access and download the data as well as implement the evaluation metrics. I have have not judged the technical aspects of DREAMTools but I note that the difficulties is installing the software experienced by Konrad Hinsen points to the fact that is it of limited usability at this moment. In summary, I believe that DREAMTools can be approved in the off chance that new DREAM challenges use the package but at the moment it is difficult to install and of little use.

I have read this submission. I believe that I have an appropriate level of expertise to confirm that it is of an acceptable scientific standard, however I have significant reservations, as outlined above.

# Response

**Published:** 2016-01-18

**Authors:** Thomas Cokelaer

**Affiliations:** EMBL European Bioinformatics Institute, UK

Dear Rafael,

First of all, thank you very much for your time in reviewing this paper.

From your experience and concerns, we'd like to clarify why DREAMTools is a valuable tools for the scientific community.

First of all, we acknowledge that the installation does not work for every platform and system; the fact is that we did not consider all platforms and systems during our development ! Even though the Python language is cross-platform by definition, it is nevertheless also linked to packages that require compilation (e.g., numpy). Although DREAMTools provides a Python API, behind the scene we also use R and Perl and C languages. Today, Python and R lead the data science but new languages (e.g., Julia) will also enter the scene. It is clear that one of the struggles many data scientists face nowadays is to design software that would work for everyone in this complex multi-language scenario. People have worked hard for the last decades without a definite solution. This does not mean we cannot and the Anaconda solution is one example of a great initiative that aim at alleviate this general issue.

We are moving towards that direction by proposing to use Anaconda to install some of the dependencies on which DREAMTools relies. Ideally, we'd like to provide also DREAMTools as an Anaconda package, which would solve lots of the problems we are currently facing.

Note that once Anaconda is installed, the DREAMTools package can be installed under Linux in a couple of minutes (See our Travis continous integration https://travis-ci.org/dreamtools/dreamtools). This is also true under Windows 7 and Mac 10.10

Coming back on DREAMTools itself and its scientific interest, we believe that DREAMTools has already been valuable. First, because it was used in a few challenges (e.g., DREAM7, parameter estimation challenge, DREAM8 HPN Breast Cancer challenge) as the base code for the scoring functions during the challenge itself and to produce results and figures in publications. Second, because it assembles data and scoring functions from old challenges (DREAM2 to DREAM6) that would not have been available otherwise. Besides, all codes in DREAM2 to DREAM6 were originally written in matlab. One of our aim was to provide open source codes, which are now available inside DREAMTools. We also do not agree with this statement:

"At the moment, any group interested in benchmarking their prediction method for a particular challenge can easily access and download the data as well as implement the evaluation metrics"

Data are accessible indeed but scoring metrics needed to be recoded and are now available thanks to the effort that have been put in  DREAMTools. Of course each group can recode its own evaluation metric but what is the point since it has been done and gathered in a single place. The idea of DREAMTools is that each group can just re-use our code since it is supported by the authors who wrote the scoring functions used in DREAM challenges !

You also wrote: "I believe that DREAMTools can be approved in the off chance that new DREAM challenges use the package". More than 15 scoring functions for 15 of the earlier DREAM challenges have been made available to the open-source community. Another 15 scoring functions for more recent challenges have been made available within a single framework factorising code in the process. We believe that new challenges will be added either by DREAM developers or members of the open-source community.

Again we thank you for your time and feedbacks.

Thomas Cokelaer on behalf of DREAMTools developers

# Response

**Published:** 2016-03-30

**Authors:** Thomas Cokelaer

**Affiliations:** EMBL European Bioinformatics Institute, UK

Dear Rafael,

For your information, DREAMTools is now available on the bioconda (https://bioconda.github.io/) channel of Anaconda (https://www.continuum.io/downloads). Installation of the latest DREAMTools release should now be easier for MAC and Linux users as explained within the online documentation (http://dreamtools.readthedocs.org/en/latest/#installation).

Best

Thomas

# Peer review: Referee response for version 1

**DOI:** 10.5256/f1000research.7664.r11288

**Published:** 2015-11-26

**Authors:** Konrad Hinsen

**Affiliations:** 1 Centre de Biophysique Moléculaire (UPR4301 CNRS), Rue Charles Sadron, Orléans, France

This article describes a software tool that implements in a transparent and reusable way the scoring of DREAM challenges, covering 80% of the challenges that have taken place. The tool thus ensures replicability of past challenges and also permits researchers to use them in evaluating their future work.

The software is fully open and well documented, conforming to the highest standards of open science. The article is clear and explains both the motivation for and the design of the software in sufficient detail.

Unfortunately I did not succeed in installing and running the software following the provided instructions (see https://github.com/dreamtools/dreamtools/issues/60 and https://github.com/dreamtools/dreamtools/issues/59 for the details). These issues seem minor, but prevent a full evaluation at this time. This is my only reason for approving this article with reservations.

I have read this submission. I believe that I have an appropriate level of expertise to confirm that it is of an acceptable scientific standard, however I have significant reservations, as outlined above.

# Response

**Published:** 2015-12-04

**Authors:** Thomas Cokelaer

**Affiliations:** EMBL European Bioinformatics Institute, UK

Dear Konrad,

First of all thanks a lot for your review and comments. 

The main issue you had was that the installation failed, which is unfortunate indeed. It appears that you tried to install DREAMTools using Python3 and we developed the software for Python2 only. The paper did not mention the Python2/3 compatibility and this may have misled you as it would misled many people. Even though we were mentionning in the README file that DREAMTools is developed for Python2 only, we fully understand that users may use Python3 by default. The rationale for sticking to Python2 was that a few librariries on which we were relying were not available for Python3 at the time we developed DREAMTools. Meanwhile, most of them have been ported to Python3 but one. Yet, we decided to port DREAMTools to Python3 since we also provide of temporary python3 version of the library that is not yet available for Python3. Consequently, DREAMTools is now available for Python2.7 and various variant of Python3.X . We hope that this should help you to further test the software.  The README/installation section have been modified to reflect those changes and guide users in the installation process.

As for the issues that you have reported https://github.com/dreamtools/dreamtools/issues/60 and https://github.com/dreamtools/dreamtools/issues/59 we believe that they should be fixed by now. (The first error was enterily related Python3 and second issue has been solved by providing informative message to the user and additional documentation).

We have released a new version of DREAMTools on pypi website and the current official release is 1.1.1

We thank again the reviewer for his time and comments that motivated us to port DREAMTools to Python3 so as to give access to the software to a wider community.

Best

Thomas Cokelaer on behalf of the DREAMTools team.

# Response

**Published:** 2015-12-08

**Authors:** Konrad Hinsen

**Affiliations:** Centre de Biophysique Moléculaire (CNRS), France

Dear Thomas,

thanks for looking into this, and working on Python 3 compatibility. I do understand that Python 3 compatibility is not trivial!

I tried once more to install dreamtools for Python 3, following the instructions, but failed again. The problem comes from a dependency (see https://github.com/dreamtools/dreamtools/issues/61), so I am not sure there is much you can do about it.

Next I tried installing dreamtools with Python 2. That fails as well, again because of a dependency (again gevent!) that fails to install correctly. That may well be specific to the MacOS platform, or to some other detail of my computational environment. I suspect there isn't much you can do about it, but it still means that I cannot test-drive dreamtools at all.

# Response

**Published:** 2015-12-08

**Authors:** Thomas Cokelaer

**Affiliations:** EMBL European Bioinformatics Institute, UK

Dear Konrad,

I'm sorry to see that the installation causes problems. The portage of DREAMTools to Python3 was done under Linux systems for different versions of Python3 (3.3, 3.4, 3.5) and tested on  Travis (https://travis-ci.org/dreamtools/dreamtools). The gevent external library may not be needed strictly speaking so we can probably find a workaround.

Another solution that we will provide very soon is to use Anaconda environment with pre-installed packages (e.g. gevent). In the next version of DREAMTools, we will provide a conda-compatible package to guarantee that the installation is possible on all systems. We have tested this option recently on another software with success.

Again sorry to hear that you cannot test the software easily and we'll work on making this possible as soon as possilble.

Regards

Thomas Cokelaer

# Response

**Published:** 2015-12-10

**Authors:** Konrad Hinsen

**Affiliations:** Centre de Biophysique Moléculaire (CNRS), France

The gevent problem I ran into under Python 2.7 turned out to be known already: https://github.com/gevent/gevent/issues/656. Since it is specific to MacOS 10.9, I tried on a machine that runs 10.10 and I managed to install dreamtools there with no(!) problems (Python 2.7, I didn't try Python 3 yet).

Unfortunately, fragile dependencies are becoming more and more of a problem in the Python universe. I wish you all the luck you need with an approach based on Anaconda - my own experiences with that approach are mixed to say the least.

To get a first experience with dreamtools, I tried running the command lines given as examples in the README. Unfortunately, I ran into serious usability issues (see https://github.com/dreamtools/dreamtools/issues/63), and in particular it seems that the example dataset is not open. Could you suggest another dataset I can work with?

# Response

**Published:** 2016-01-18

**Authors:** Thomas Cokelaer

**Affiliations:** EMBL European Bioinformatics Institute, UK

Dear Konrad,

Having a Python software that runs on all platforms/systems is a challenge by itself ! The issue with gevent on MacOS 10.9 you got is an example, which is not unique. This is a general issue for the scientific community and as you pointed out both for users and developers. I think Anaconda does help a lot. I have started to use Anaconda only recently but I see a great value for future development even though not all problems are fixed. So, we decided to provide a solution based on Anaconda for DREAMTools. We do not yet provide a pre-compiled version of DREAMTools within anaconda.org but this may be the best solution for future versions. For now,  DREAMTools is still downloaded from Pypi but most dependencies (e.g., numpy) can be obtained as pre-compiled version from Anaconda packages.

As for the problems you reported in the issue 63 on github, I fully appreciate your concerns and the API and examples have been updated to make user's experience a bit better. One of the major issue was that the example in the documentation failed because synapse expected the condition of use (of the data) to be accepted inside the browser. So, the code was changed to tell the user what to do in this situation.

Other cryptic messages and issues reported in the issue 63 should have been addressed as well.

We also suggest another challenge in the examples, which do not require access to synapse (challenge D6C3).

Thomas Cokelaer

# Response

**Published:** 2016-03-30

**Authors:** Thomas Cokelaer

**Affiliations:** EMBL European Bioinformatics Institute, UK

Dear Konrad,

For your information, DREAMTools is now available on the bioconda (https://bioconda.github.io/) channel of Anaconda (https://www.continuum.io/downloads). Installation of the latest DREAMTools release should now be easier for MAC and Linux users as explained within the online documentation (http://dreamtools.readthedocs.org/en/latest/#installation).

Best

Thomas
